# Management of early-stage triple-negative breast cancer: recommendations of a panel of experts from the Brazilian Society of Mastology

**DOI:** 10.1186/s12885-022-10250-x

**Published:** 2022-11-22

**Authors:** Ruffo Freitas-Junior, Vilmar Marques de Oliveira, Antonio Luiz Frasson, Francisco Pimentel Cavalcante, Fabio Postiglione Mansani, André Mattar, Felipe Pereira Zerwes, Adriana Magalhães de Oliveira Freitas, Alessandra Borba Anton de Souza, Andrea P. Damin, Annamaria Massahud Rodrigues dos Santos, Carlos Alberto Ruiz, Clécio Ênio Murta de Lucena, Eduardo Camargo Millen, Fábio Bagnoli, Felipe Andrade, Frank Lane Braga Rodrigues, Gil Facina, Guilherme Novita, Jose Luiz Pedrini, José Pereira Guará, Leonardo Ribeiro Soares, Marcus Vinicius de Nigro Corpa, Mauro Passos, Nancy Cristina Ferraz de Lucena Ferreira, Nilceana Maya Aires Freitas, Rafael Henrique Szymanski Machado, Roberto Kepler da Cunha Amaral, Tomás Reinert, Vinicius Milani Budel

**Affiliations:** 1 Advanced Center for Breast Diagnosis (CORA), Federal University of Goiás and the Araújo Jorge Hospital, Goiás Association for the Combat of Cancer, 1ª Avenida, s/n, Setor Universitário, Goiânia, GO 74605-050 Brazil; 2 School of Medical Sciences, Santa Casa de Misericórdia de São Paulo, São Paulo, SP Brazil; 3 Pontifical Catholic University of Rio Grande do Sul, Porto Alegre (RS), Brazil and the Hospital Israelita Albert Einstein, São Paulo, SP Brazil; 4 Fortaleza General Hospital, Fortaleza, CE Brazil; 5 Department of Medicine, State University of Ponta Grossa, Ponta Grossa, PR Brazil; 6 Reference Center in Women’s Health Care, Pérola Byington Hospital, São Paulo, SP Brazil; 7 Pontifical Catholic University of Rio Grande do Sul, Porto Alegre, RS Brazil; 8 Larmony Clinic, Florianópolis, SC Brazil; 9 Federal University of Rio Grande do Sul, Porto Alegre, RS Brazil; 10 Santa Casa de Misericórdia de Belo Horizonte, Belo Horizonte, MG Brazil; 11 Teaching Hospital, School of Medicine, University of São Paulo, São Paulo, SP Brazil; 12 Federal University of Minas Gerais, Belo Horizonte, MG Brazil; 13 Oncoclínicas Institute, Rio de Janeiro, RJ Brazil; 14 School of Medical Sciences, Santa Casa de Misericórdia de São Paulo and the Hospital Israelita Albert Einstein, São Paulo, SP Brazil; 15 Department of Breast Surgery, Hospital Sírio-Libanês, São Paulo, SP Brazil; 16 Institute of Mastology and Oncology and the Goiania Institute of Hematology (INGOH), Goiânia, GO Brazil; 17 Department of Gynecology, Federal University of São Paulo, São Paulo, SP Brazil; 18 Hospital Israelita Albert Einstein, São Paulo, SP Brazil; 19 Nossa Senhora da Conceição Hospital, Porto Alegre, RS Brazil; 20 Teaching Hospital of the Federal University of Maranhão, São Luís, MA Brazil; 21 Advanced Center for Breast Diagnosis (CORA), Federal University of Goiás and the Dona Iris Women’s and Maternity Hospital, Goiânia, GO Brazil; 22 Department of Pathology, Hospital Israelita Albert Einstein, São Paulo, SP Brazil; 23 Hospital de Base, Federal District, Brasília, DF Brazil; 24 Barão de Lucena Hospital, Federal University of Pernambuco, Recife, PE Brazil; 25 Radiotherapy Unit, Araújo Jorge Cancer Hospital, Goiás Association for the Combat of Cancer, and Brazilian Center for Radiotherapy, Oncology and Mastology (CEBROM), Goiânia, GO Brazil; 26 Hospital Federal da Lagoa, Rio de Janeiro, RJ Brazil; 27 Grupo CAM / Oncoclínicas, Salvador, BA Brazil; 28 Oncoclínicas Porto Alegre, Porto Alegre (RS) and the Serra Gaúcha Research Center, Caxias do Sul, RS Brazil; 29 Teaching Hospital of the Federal University of Paraná, Curitiba, PR Brazil

**Keywords:** Breast cancer, Triple-negative breast cancer, Consensus development conferences

## Abstract

**Background:**

Triple-negative breast cancer (TNBC) is a heterogenous subtype involving different patterns of behavior and clinical course, demanding a complex, individualized sequence of treatment. The knowledge and attitudes of the affiliated members of the Brazilian Society of Mastology regarding TNBC were evaluated and a consensus regarding management and treatment was reached.

**Methods:**

Affiliates completed a survey involving 44 objective questions. In addition, a specialist meeting was held with 27 experts and 3 ad hoc consultants. The panelists completed the survey before and after brainstorming. Answers achieving 70% of agreement were considered consensual. The chi-square test was used to compare answers between panelists and affiliates and the Kappa coefficient to calculate agreement.

**Results:**

Consensus among the panelists increased from 26 (59.1%) to 32 questions (72.7%) following brainstorming (*p* = 0.17), including 7/10 questions on systemic treatment. Among the affiliates, consensus was achieved for 24 questions (54.5%), resulting in moderate agreement (κ = 0.445). Neoadjuvant chemotherapy should be indicated for almost all cases (except cT1a-b N0) and should include platinum agents. When indicated, immunotherapy is part of the standard of care. The panel reaffirmed the concept of *no ink on tumor* as indicative of adequate margins and the possibility of sentinel lymph node biopsy for cN1 patients who become cN0 following neoadjuvant therapy. Controversies remain on combining immunotherapy with capecitabine/olaparib in pertinent cases.

**Conclusion:**

Expert consensus was achieved for > 70% of the questions, with moderate agreement between panelists and affiliates. Educational interventions on systemic breast cancer treatment affected decision-making in 60% of the questions.

**Supplementary Information:**

The online version contains supplementary material available at 10.1186/s12885-022-10250-x.

## Introduction

Triple-negative breast cancer (TNBC) is immunohistochemically defined as lacking expression of estrogen and progesterone receptors (ER, PR) and absence of over-expression of human epidermal growth factor receptor 2 (HER-2), and represents 10-20% of all cases of the disease [1, 2]. This heterogenous subtype involves different behaviors and clinical courses resulting from different profiles of gene expression and molecular biology [3, 4]. Specific medical knowledge is critical for adequate diagnosis, management and treatment of TNBC.

TNBC treatment has become progressively more complex and individualized [5]. In neoadjuvant therapy (NAT), combining pembrolizumab and chemotherapy improved pathologic complete response (pCR) and event-free survival (EFS), modifying clinical practice [6, 7]. Likewise, platinum agents [8] and poly adenosine diphosphate-ribose polymerase (PARP) inhibitors have been used in women with germline mutations in the *BRCA1* and *BRCA2* genes [9]. Controversies remain regarding clipping of axillary lymph nodes, sentinel lymph node biopsy (SLNB) following neoadjuvant chemotherapy (NAC), staging and initial disease management [5, 10, 11].

The Brazilian Society of Mastology (SBM) provides updated information and continued education to its affiliates [12] with a view to reducing breast cancer-related morbidity and mortality in the country [13, 14]. Nevertheless, the knowledge and attitudes of this Brazilian medical population may still reflect those of low-and-middle-income countries.

This study evaluated the knowledge and attitudes of SBM members regarding TNBC and drew up consensus guidelines on the diagnosis, management and treatment of the disease.

## Methods

An email survey (December 2021 to January 2022) was sent to all 1400 active SBM members. All fully completed questionnaires were included in the analysis.

The 44-item questionnaire on the management and treatment of TNBC was developed using SurveyMonkey. Each question had three/four alternative answers, 28/44 being on a Likert-type scale. The American Association for Public Opinion Research recommendations were followed [15].

The demographic data surveyed included: the respondent’s sex; board-certification as a specialist in breast disease (yes/no), the geographical region of residence/work; and whether the respondent worked in an academic institute. Questions on TNBC diagnosis and locoregional treatment involved immunohistochemistry, staging, clipping of axillary lymph nodes, axillary management and post-mastectomy radiotherapy. Questions on systemic treatment focused particularly on NAT and immunotherapy. For standardization purposes, the questions considered patients with tumors over 1.0 cm in diameter, in good general health and with a life expectancy > 10 years.

A meeting of 27 experts was held on December 3, 2021 during a Breast Cancer Congress in Gramado, RS, Brazil, which also included 3 ad hoc consultants from the Brazilian Societies of Clinical Oncology, Pathology, and Radiotherapy, respectively. Only the breast surgeons, however, were allowed to vote.

The panelists answered the questions in the survey according to the Delphi method [16]. The answers reaching 70% of agreement were considered consensual. The questions for which no consensus was reached were then discussed based on a literature review (brainstorming session). Subsequently, a further round of voting was held for the 44 questions. Double consensus was defined as when agreement was > 70% in the first round of responses from the panelists and in the independent voting by the affiliates.

### Ethical issues

The SBM review board approved the study protocol. Returning a completed questionnaire implied agreement to participate in the study and the consent to publish was obtained from all participants. The study procedures were conducted in compliance with current Brazilian legislation and the 1964 Helsinki Convention.

### Statistical analysis

SPSS, version 26.0 (IBM Corporation, Armonk, NY) was used throughout the analysis. Answers were described as absolute (n) or relative frequencies (%). Pearson’s chi-square test was used to compare the answers given by the panelists before and after brainstorming, and to compare the initial panel answers with those given by the affiliates. A post hoc analysis was performed whenever pertinent [17]. Significance was set at *p* < 0.05.

The kappa coefficient was used to calculate agreement between the panelists and the affiliates. Agreement was defined as poor (κ < 0.00), slight (0.0 ≤ κ ≤ 0.2), fair (0.21 ≤ κ ≤ 0.4), moderate (0.41 ≤ κ ≤ 0.6), substantial (0.61 ≤ κ ≤ 0.8) or almost perfect (0.81 ≤ κ ≤ 1.0) [18].

## Results

Mean age of the panelists was 51.7 ± 9.7 years. Most (81.5%) were male, 44.4% lived/worked in the southeast of the country, 96.3% lived in state capital cities and 92.6% were associated with academic institutions. The mean age of the 214 affiliates who completed the survey was 46.1 years (*p* = 0.05), 50.9% were female (*p* = 0.01) and 29.4% did not live in state capital cities (Additional file 1: Table S1).

Among the panelists, consensus was achieved for 26 questions (59.1%), increasing to 32 questions (72.7%) after brainstorming (*p* = 0.17) (κ = 0.465) (Additional file 1: Table S2, Additional file 2: Table S2, Additional file 3: Table S3, Additional file 4: Table S4). Disagreement was > 90% for three items in the survey: i) radiological evaluation of flap thickness following nipple-sparing mastectomy (NSM); ii) bilateral mastectomy for patients with the wild-type *BRCA*; and iii) radiotherapy following mastectomy in stage T1N0.

Among the affiliates, consensus was achieved for 24 questions (54.5%) (Additional file 5: Table S5). There was moderate agreement (κ = 0.445) and no significant difference (*p* = 0.661) in relation to the panelists prior to brainstorming. Tables 1, 2, 3 and 4 describe the agreement between panelists and affiliates, with differences being significant for nine questions (five related to systemic treatment) (Table 5).

**Table 1:** Agreement between the panelists and the SBM affiliated breast surgeons for the questions associated with diagnosis

Questions	Panelists before brainstorming		Affiliated breast surgeons	
	Disagreement n (%)	Agreement n (%)	Disagreement n (%)	Agreement n (%)
**Q01.** HER2-negative patients with hormone receptor expression < 10% at IHC should be treated as TNBC.	11 (40.7)	16 (59.3)	81 (37.9)	133 (62.1)
**Q02.** HER2-negative patients with hormone receptor-positive expression at IHC, but with gene signature suggestive of the basal-like subtype should be treated as TNBC.	11 (40.7)	16 (59.3)	50 (23.4)	164 (76.6)
**Q03.** Axillary ultrasound should be performed as routine in patients with clinically negative axilla.	10 (37.0)	17 (63.0)	73 (34.1)	141 (65.9)
**Q04.** MRI of the breasts should be recommended as routine at surgical planning.	21 (77.8)	6 (22.2)	134 (62.6)	80 (37.4)
**Q05.** Gene panel testing should be recommended for all cases.	8 (29.6)	19 (70.4)	85 (39.7)	129 (60.3)
**Q06.** Systemic staging should be recommended as routine for asymptomatic patients.	4 (14.8)	23 (85.2)	17 (7.9)	197 (92.1)
**Q20.** In T1/2 N0 patients submitted to mastectomy, evaluation of flap thickness using imaging should be recommended following surgery.	25 (92.6)	2 (7.4)	146 (68.2)	68 (31.8)
**Q28.** In patients to be submitted to upfront surgery with BCS, “*no ink on tumor*” should be considered indicative of adequate margins.	1 (3.7)	26 (96.3)	36 (16.8)	178 (83.2)
**Q29.** In patients to be submitted to BCS following neoadjuvant chemotherapy, “*no ink on tumor*” should be considered indicative of adequate margins.	2 (7.4)	25 (92.6)	41 (19.2)	173 (80.8)
**Q30.** Patients who are candidates for BCS and who will undergo neoadjuvant chemotherapy should receive some kind of tumor marker (skin pigmentation or clipping or iodine-125 seed implantation on the tumor).	3 (11.1)	24 (88.9)	54 (25.2)	160 (74.8)
**Q31.** Imaging tests should be recommended following neoadjuvant treatment to evaluate response.	1 (3.7)	26 (96.3)	6 (2.8)	208 (97.2)
**Q33.** Following neoadjuvant treatment, staging tests should be requested again.	8 (29.6)	19 (70.4)	75 (35.0)	139 (65.0)
**Q34.** Further IHC should be requested after neoadjuvant treatment if there is residual disease.	7 (25.9)	20 (74.1)	91 (42.5)	123 (57.5)

*SBM* Brazilian Society of Mastology, *TNBC* triple-negative breast cancer, *IHC* immunohistochemistry, *BCS* breast-conserving surgery, *MRI* magnetic resonance imaging

**Table 2:** Agreement between the panelists and the SBM affiliated breast surgeons for the questions related to surgical treatment

Question	Panelists before brainstorming		Affiliated breast surgeons	
	Disagreement n (%)	Agreement n (%)	Disagreement n (%)	Agreement n (%)
**Q07.** If the axilla is clinically positive, neoadjuvant chemotherapy should be recommended.	0 (0.0)	27 (100.0)	7 (3.3)	207 (96.7)
**Q08.** If the axilla is clinically negative, when conservative treatment is possible, neoadjuvant chemotherapy should be recommended for tumors > 1.0 cm.	10 (37.0)	17 (63.0)	98 (45.8)	116 (54.2)
**Q09.** Following BCS and when the SLN is positive at upfront surgery, in addition to systemic treatment, radiotherapy should be recommended as local treatment.	5 (18.5)	22 (81.5)	58 (27.1)	156 (72.9)
**Q10.** Following mastectomy and when the SLN is positive at upfront surgery, in addition to systemic treatment, radiotherapy should be recommended as local treatment.	5 (18.5)	22 (81.5)	95 (44.4)	119 (55.6)
**Q11.** If the SLN is positive following neoadjuvant therapy, ALND should be recommended.	4 (14.8)	23 (85.2)	35 (16.4)	179 (83.6)
**Q12.** If the axilla is initially positive, some form of lymph node marking should be recommended prior to neoadjuvant therapy.	19 (70.4)	8 (29.6)	113 (52.8)	101 (47.2)
**Q13.** In patients with positive axilla who achieve complete clinical response to neoadjuvant therapy with negative SLN not previously marked, ALND should be recommended in all cases.	26 (96.3)	1 (3.7)	196 (91.6)	18 (8.4)
**Q14.** If germline genetic testing is negative, bilateral mastectomy should be recommended.	27 (100.0)	0 (0.0)	207 (96.7)	7 (3.3)
**Q16.** If germline genetic testing is negative, the patient’s family history should be considered when recommending bilateral mastectomy.	7 (25.9)	20 (74.1)	73 (34.1)	141 (65.9)
**Q17.** If testing for high-penetrance gene mutations is positive, the possibility of bilateral mastectomy should be considered.	0 (0.0)	27 (100.0)	4 (1.9)	210 (98.1)
**Q19.** If the NAC is disease-free and testing for high-penetrance gene mutations is positive, nipple-sparing mastectomy should be recommended.	0 (0.0)	27 (100.0)	5 (2.3)	209 (97.7)
**Q27.** If testing for high-penetrance gene mutations is positive, BCS should be considered sufficient.	17 (63.0)	10 (37.0)	129 (60.3)	85 (39.7)

*SBM* Brazilian Society of Mastology, *BCS* breast-conserving surgery, *SLN* sentinel lymph node, *ALND* axillary lymph node dissection, *NAC* nipple-areola complex

**Table 3:** Agreement between the panelists and the SBM affiliated breast surgeons for the questions related to radiotherapy

Questions	Panelists before brainstorming		Affiliated breast surgeons	
	Disagreement n (%)	Agreement n (%)	Disagreement n (%)	Agreement n (%)
**Q21.** In T1N0 patients submitted to simple mastectomy (removal of the NAC), radiotherapy of the thoracic wall should be recommended as routine.	26 (96.3)	1 (3.7)	201 (93.9)	13 (6.1)
**Q22.** In T2N0 patients submitted to simple mastectomy (removal of the NAC), radiotherapy of the thoracic wall should be recommended as routine.	18 (66.7)	9 (33.3)	156 (72.9)	58 (27.1)
**Q23.** In T1/2 N0 patients submitted to nipple-sparing mastectomy, radiotherapy of the thoracic wall should be recommended as routine.	22 (81.5)	5 (18.5)	161 (75.2)	53 (24.8)
**Q24.** In T1/2 N1 patients submitted to mastectomy who have achieved pCR following neoadjuvant therapy, radiotherapy of the thoracic wall should be recommended.	0 (0.0)	27 (100.0)	74 (34.6)	140 (65.4)
**Q25.** In T1/2 N2 patients submitted to mastectomy who have achieved pCR following neoadjuvant therapy, radiotherapy of the thoracic wall should be recommended.	0 (0.0)	27 (100.0)	25 (11.7)	189 (88.3)
**Q26.** In T3N0 patients submitted to mastectomy who have achieved pCR following neoadjuvant therapy, radiotherapy of the thoracic wall should be recommended.	0 (0.0)	27 (100.0)	21 (9.8)	193 (90.2)

*SBM* Brazilian Society of Mastology, *NAC* nipple-areola complex, *pCR* pathologic complete response

**Table 4:** Agreement between the panelists and the SBM affiliated breast surgeons for the questions related to systemic treatment

Questions	Panelists before brainstorming		Affiliated breast surgeons	
	Disagreement n (%)	Agreement n (%)	Disagreement n (%)	Agreement n (%)
**Q35.** In patients with no *BRCA* germline mutation, platinum agents should be recommended in neoadjuvant treatment.	11 (40.7)	16 (59.3)	105 (49.1)	109 (50.9)
**Q36.** In patients with the *BRCA* germline mutation, the use of platinum agents in neoadjuvant treatment should be recommended.	11 (40.7)	16 (59.3)	39 (18.2)	175 (81.8)
**Q37.** In patients who will be submitted to neoadjuvant treatment, the addition of immunotherapy should be recommended as routine.	13 (48.1)	14 (51.9)	149 (69.6)	65 (30.4)
**Q38.** In patients who will be submitted to neoadjuvant treatment, PD-L1 status should be taken into consideration when recommending immunotherapy.	14 (51.9)	13 (48.1)	63 (29.4)	151 (70.6)
**Q39.** *BRCA* status should play a role in the decision regarding whether to recommend neoadjuvant treatment with immunotherapy.	22 (81.5)	5 (18.5)	120 (56.1)	94 (43.9)
**Q40.** In patients who will be submitted to neoadjuvant treatment with immunotherapy, dose-dense anthracycline-based chemotherapy should be used.	7 (25.9)	20 (74.1)	53 (24.8)	161 (75.2)
**Q41.** In patients with no *BRCA* germline mutation submitted to neoadjuvant treatment with immunotherapy and who achieve pCR, immunotherapy should be continued during adjuvant therapy.	11 (40.7)	16 (59.3)	116 (54.2)	98 (45.8)
**Q42.** In patients with no *BRCA* germline mutation submitted to neoadjuvant treatment with immunotherapy and who have residual disease, immunotherapy should be continued during adjuvant therapy.	7 (25.9)	20 (74.1)	62 (29.0)	152 (71.0)
**Q43.** In patients with no *BRCA* germline mutation submitted to neoadjuvant therapy with immunotherapy and in whom there is residual disease, the use of adjuvant immunotherapy associated with capecitabine should be suggested.	13 (48.1)	14 (51.9)	54 (25.2)	160 (74.8)
**Q44.** In patients with the *BRCA* germline mutation submitted to neoadjuvant therapy with immunotherapy and who achieve pCR, the use of adjuvant immunotherapy associated with olaparib should be suggested.	18 (66.7)	9 (33.3)	104 (48.6)	110 (51.4)

*SBM* Brazilian Society of Mastology, *pCR* pathologic complete response

**Table 5:** The nine questions for which the proportion of answers differed significantly between the panelists and the SBM affiliated breast surgeons

Questions	Groups		***p***-value*
	Panelists before brainstorming n (%)	Affiliated breast surgeons n (%)	
**Q09.** Following BCS and when the SLN is affected at upfront surgery, in addition to systemic treatment would you recommend:			
Axillary dissection?	1 (3.7)	47 (22.0)^a^	**0.02**
Watchful waiting?	4 (14.8)^a^	11 (5.1)	
Radiotherapy?	22 (81.5)	156 (72.9)	
**Q10.** In a patient submitted to mastectomy with positive SLN at upfront surgery, in addition to systemic treatment what would you recommend:			
Axillary dissection?	4 (14.8)	86 (40.2)	**0.03**
Watchful waiting?	1 (3.7)	9 (4.2)	
Radiotherapy?	22 (81.5)	119 (55.6)	
**Q20.** In T1/2 N0 patients submitted to mastectomy, evaluation of flap thickness using imaging should be recommended following surgery.			
I agree	2 (7.4)	68 (31.8)	**0.01**
I disagree	25 (92.6)	146 (68.2)	
**Q24.** In T1/2 N1 patients submitted to mastectomy who have achieved pCR following neoadjuvant treatment, radiotherapy of the thoracic wall should be recommended.			
I agree	27 (100.0)	140 (65.4)	**0.01**
I disagree	0 (0.0)	74 (34.6)	
**Q36.** In patients with the *BRCA* germline mutation, the use of platinum agents in neoadjuvant treatment should be recommended.			
I agree	16 (59.3)	175 (81.8)	**0.01**
I disagree	11 (40.7)	39 (18.2)	
**Q37.** In patients who will be submitted to neoadjuvant treatment, the addition of immunotherapy should be recommended as routine.			
I agree	14 (51.9)	65 (30.4)	**0.02**
I disagree	13 (48.1)	149 (69.6)	
**Q38.** In patients who will be submitted to neoadjuvant treatment, PD-L1 status should be taken into consideration when recommending immunotherapy.			
I agree	13 (48.1)	151 (70.6)	**0.02**
I disagree	14 (51.9)	63 (29.4)	
**Q39.** *BRCA* status should play a role in the decision regarding whether to recommend neoadjuvant treatment with immunotherapy.			
I agree	5 (18.5)	94 (43.9)	**0.01**
I disagree	22 (81.5)	120 (56.1)	
**Q43.** In patients with no *BRCA* germline mutation submitted to neoadjuvant therapy with immunotherapy and in whom there is residual disease, the use of adjuvant immunotherapy associated with capecitabine should be suggested.			
I agree	14 (51.9)	160 (74.8)	**0.01**
I disagree	13 (48.1)	54 (25.2)	

*SBM* Brazilian Society of Mastology, *BCS* breast-conserving surgery, *SLN* sentinel lymph node, *pCR* pathologic complete response

*Chi-square test; ^a^Post hoc; n = absolute frequency; % = relative frequency

The panelists’ answers before and after brainstorming are shown in Additional file 6: Tables S6.1–S6.4. Consensus was achieved or modified following brainstorming for 7/10 questions on systemic treatment (Table 6) and for one question related to surgical treatment. Additional file 7: Tables S7.1–S7.4 describe the degree of consensus between panelists and affiliates. Finally, the main consensus recommendations are summarized in Tables 7 and 8, and the main controversies in Table 9.

**Table 6:** Responses of the panelists before and after brainstorming for the questions for which there was change in consensus

Questions	Before brainstorming		After brainstorming		***p***-value*
	Disagreement ***n*** (%)	Agreement ***n*** (%)	Disagreement ***n*** (%)	Agreement ***n*** (%)	
**Q05.** Gene panel testing should be recommended for all cases.	8 (29.6)	19 (70.4)	10 (37.0)	17 (63.0)	0.56
**Q09.** Following BCS and when the SLN is positive at upfront surgery, in addition to systemic treatment, radiotherapy should be recommended as local treatment.	5 (18.5)^a^	22 (81.5)	11 (40.7)	16 (59.3)	**0.02**
**Q22.** In T2N0 patients submitted to simple mastectomy (removal of the NAC), radiotherapy of the thoracic wall should be recommended as routine.	18 (66.7)	9 (33.3)	21 (77.8)	6 (22.2)	0.36
**Q33.** Following neoadjuvant treatment, staging exams should be requested again only in the case of partial response or progression.	9 (33.3)	18 (66.7)	8 (29.6)	19 (70.4)	0.95
**Q35.** In patients with no *BRCA* germline mutation, platinum agents should be recommended in neoadjuvant treatment.	11 (40.7)	16 (59.3)	7 (25.9)	20 (74.1)	0.24
**Q36.** In patients with the *BRCA* germline mutation, the use of platinum agents in neoadjuvant treatment should be recommended.	11 (40.7)	16 (59.3)	5 (18.5)	22 (81.5)	0.07
**Q37.** In patients who will be submitted to neoadjuvant treatment, the addition of immunotherapy should be recommended as routine.	13 (48.1)	14 (51.9)	5 (18.5)	22 (81.5)	**0.02**
**Q38.** In patients who will be submitted to neoadjuvant treatment, PD-L1 status should be taken into consideration when recommending immunotherapy.	14 (51.9)	13 (48.1)	22 (81.5)	5 (18.5)	**0.02**
**Q40.** In patients who will be submitted to neoadjuvant treatment with immunotherapy, dose-dense anthracycline-based chemotherapy should be used.	7 (25.9)	20 (74.1)	15 (55.6)	12 (44.4)	**0.03**
**Q41.** In patients with no *BRCA* germline mutation submitted to neoadjuvant treatment with immunotherapy and who achieve pCR, immunotherapy should be continued during adjuvant therapy.	11 (40.7)	16 (59.3)	3 (11.1)	24 (88.9)	**0.01**
**Q44.** In patients with the *BRCA* germline mutation submitted to neoadjuvant therapy with immunotherapy and who achieve pCR, the use of adjuvant immunotherapy associated with olaparib should be suggested.	18 (66.7)	9 (33.3)	8 (29.6)	19 (70.4)	0.77

*BCS* breast-conserving surgery, *SLN* sentinel lymph node, *NAC nipple-*areolar *complex, pCR* pathologic complete response

*Chi-squared test; ^a^Post hoc; n = absolute frequency; % = relative frequency

**Table 7:** Summary of main recommendations and how can practice change if they are applied

	Summary of recommendations	How can practice change?
	Evaluate genetic testing for most TNBC cases.	- Genetic counseling for patients and families. - Possibility of risk-reducing therapies. - Individualized treatment according to the presence of pathogenic mutations.
	Individualize axillary ultrasound according to potential for treatment change.	- Avoid ALND increase in candidate patients for the ACOSOG Z0011 study. - Support the indication of adjuvant RT in cases of NAT (pN+).
	Individualize breast MRI according to potential for treatment change.	- Better surgical indication and reduction of compromised margins and re-operations. - Prioritize in the context of NAT or when surgical planning remains uncertain. - Avoid mastectomy in patients who are candidates for BCS.
	NAT for cN+ patients and cN0 patients with tumors larger than 1.0 cm.	- Tumor size reduction and possibility of more conservative surgery. - In-vivo assessment of response to treatment (chemosensitivity). - Possibility of adjuvant capecitabine for patients with residual disease.
	Perform some tumor marking before NAT.	- Appropriate surgical approach. - Reduction of compromised margins and re-operations.
	Evaluate the tumor response after NAT with breast imaging exams.	- Appropriate surgical approach. - Reduction of compromised margins and re-operations.

*ALND* axillary lymph node dissection, *BCS* breast-conserving surgery, *MRI* magnetic resonance imaging, *NAT* neoadjuvant therapy, *TNBC* triple-negative breast cancer

**Table 8:** Summary of main recommendations and how can practice change if they are applied

	Summary of recommendations	How can practice change?
	Avoid ALND for patients undergoing upfront surgery who had a positive SLNB (cT1-2).	- Decreased unnecessary ALND and surgery-related morbidity (eg, lymphedema). - Locoregional control through radiotherapy and adjuvant systemic therapy.
	Consider ALND for patients who had positive SLNB after NAT.	- More appropriate axillary staging and prognostic information about the disease. - Reduction of locoregional and distant recurrences.
	Avoid the unrestricted indication of bilateral mastectomy.	- Reduction of morbidity and financial costs related to bilateral mastectomy. - Prioritize patients with pathogenic mutations, especially if < 60 years of age.
	Consider “*no ink on tumor*” as being indicative of adequate surgical margins.	- Decreased re-operations and mastectomies by close margins.
	Avoid the evaluation of flap thickness using imaging methods.	- Avoid unnecessary RT in women with T1-2 N0 tumors submitted to mastectomy.
	Add platinum agents to NAT regimens (irrespective of *BRCA* mutations).	- Increase in pCR rate and event-free survival. - Slight increase in toxicity.
	Add immune checkpoint inhibitors to NAT (irrespective of PD-L1 expression).	- Use of pembrolizumab as indicated in the KEYNOTE-522 study. - Increase in pCR rate and event-free survival. - Slight increase in toxicity and immune-mediated events.
	Consider adjuvant capecitabine to patients undergoing NAT with residual disease.	- Increased disease-free survival and overall survival. - Slight increase in toxicity.

*ALND* axillary lymph node dissection, *BCS* breast-conserving surgery, *NAT* neoadjuvant therapy, *pCR* pathologic complete response, *RT* radiotherapy, *SLN* sentinel lymph node, *TNBC* triple-negative breast cancer

**Table 9:** Main remaining points of controversy and what additional data is needed to clarify them

Points of controversy	What additional data is needed?
Diagnosis and treatment of HER2-negative tumors and low-ER immunohistochemical expression (1-10%).	- Advancement and standardization of immunohistochemistry analysis. - Prospective studies evaluating the difference in treatment and oncological outcomes.
Treatment of ER+/HER2- tumors with a gene signature suggestive of the basal-like subtype.	- Expanding access to molecular tests. - Prospective studies evaluating the difference in treatment and oncological outcomes.
Axillary surgery for patients with initially cN+ and complete clinical response following NAT.	- Is the marking of the compromised lymph node essential (prior to NAT)? - Does the increase in the false negative rate affect the recurrence rate or overall survival? - New randomized studies evaluating clinical outcomes according to different surgical strategies.
Combination of capecitabine and pembrolizumab in patients with wild-type *BRCA* who had received NAT and had residual disease.	- New studies evaluating the efficacy and safety of this combination in the adjuvant setting.
Combination of olaparib and immunotherapy for women with the *BRCA* mutation who achieved pCR following NAT.	- New studies evaluating the efficacy and safety of this combination in the adjuvant setting.

*NAT* neoadjuvant therapy, *pCR* pathologic complete response

## Discussion and consensus

### Participants

The panelists were nationally recognized experts in TNBC treatment. Most lived in major cities and worked in academic institutions. The current transition in the profile of Brazilian breast surgeons from being predominantly male in the past, as reflected in the panelists, to a female majority now, as reflected in the affiliates, may explain the gender differences between these two groups. The poor response rate found (15.5% of the affiliates) may be due to the questionnaire’s length and its items unrelated to surgery, as well as to the high number of SBM surveys performed recently.

We considered the sampling as a limitation of our study. However, we were satisfied with 15.5% response from affiliates because their answers enriched the debate together with the panelist experts. We consider that the Brazilian surgeons are initiating the participations in surveys [13, 14]. We also need to encourage breast surgeons to discuss unclear breast cancer management topics through surveys and discussions, educational events and production of national guidelines.

### Pathology

In HER2-negative tumors, low-ER immunohistochemical expression (1-10%) still provokes controversy regarding diagnosis and treatment. Although representing < 5% of hormone-receptor-positive tumors, prognosis is poorer and the tumor often behaves similarly to TNBC [19]. Likewise, no consensus was reached regarding the treatment of ER+/HER2- tumors with a gene signature suggestive of the basal-like subtype. This concept became particularly important since no benefit in progression-free survival was found with the addition of ribociclib to standard endocrine therapy for patients with the basal-like Prediction Analysis of Microarray 50-gene set (PAM50) subtypes [20]. Nevertheless, due to the small sample size (*n* = 30) and the retrospective design of that analysis, further studies are required.

There was consensus regarding the need for further immunohistochemistry following NAT in cases of residual disease. Indeed, results can differ in relation to the percutaneous biopsy material in up to 5% of cases [21, 22]; therefore, confirming findings may significantly change the patient’s treatment and prognosis.

### Diagnostic tests

Panelists and affiliates disagreed regarding axillary ultrasonography at the time of diagnosis. Although helpful in defining the need for adjuvant radiotherapy, this may have increased axillary lymph node dissection (ALND) rather than SLNB [23]. The panelists suggested individualizing axillary ultrasonography according to the potential for a change in treatment. Avoiding magnetic resonance imaging (MRI) was a double consensus. During brainstorming, MRI was prioritized in the context of NAT or when surgical planning remains uncertain.

Nineteen panelists (70.4%) initially agreed on requesting genetic testing for all TNBC cases. Following brainstorming, this number fell to 17 (63.0%). Although some international guidelines have already recommended the test irrespective of age [24], the Brazilian Supplementary Health Agency still restricts it to patients ≤60 years of age [25]. While 48.1% of affiliates would perform systemic staging in all asymptomatic women, 48.1% of panelists prioritized investigation from anatomic stage II onwards.

### Nat

There was consensus regarding NAT for patients with clinically positive axilla, with agreement reaching 100.0% among the panelists. For patients with disease-free axilla, both the panelists (63.0%) and the affiliates (54.2%) recommended 1.0 cm as the cut-off tumor size for indicating NAT (no consensus).

In patients scheduled to undergo NAT and breast-conserving surgery (BCS), there was double consensus concerning clipping of the tumor or implantation of a radioactive iodine seed. If these techniques are unavailable, pigmentation of the skin over the tumor is also a viable option as indicated by > 20% of the affiliates. In fact, the absence of any marking on the tumor bed may prove detrimental for later surgical localization and could increase local recurrence rates [26]. Most panelists (70.4%) disagreed with the need for marking the axillary lymph node prior to NAT.

Following NAT, > 95.0% of the affiliates would request imaging tests to evaluate tumor response. However, the combination of mammography and MRI was indicated by 48.1% of the panelists and 60.3% of the affiliates (no consensus). Despite its poorer accuracy, ultrasonography can also be useful if MRI is unavailable [27]. If the disease had progressed, around 60.0% of the affiliates would also indicate further systemic staging.

### Axillary surgery

Radiotherapy for locoregional control was indicated for patients undergoing upfront surgery who had a positive SLNB (double consensus). However, despite evidence in the literature [13, 28, 29], four panelists (14.8%) and 22% of the affiliates would still indicate ALND for patients with early disease and positive SLNB. If mastectomy were required, 40.2% of affiliates would recommend ALND. Likewise, in another SBM survey, 26% of respondents failed to apply the results of the ACOSOG Z0011 study to women with TNBC, particularly breast surgeons > 50 years of age, those not associated with academic institutions, and not board-certified [13].

ALND was indicated for patients undergoing BCS who had positive SLNB after NAT (double consensus). There was no consensus regarding ALND for SLNB-negative patients with initially positive axilla (without marking) and complete clinical response following NAT. Most affiliates would avoid ALND, while 30.0% would recommend it if < 3 lymph nodes were identified at SLNB. This difference corroborates another survey in which 63.7% of Brazilian surgeons would not take molecular profiling into consideration when defining axillary management following NAT [14].

### Breast surgery

In women with TNBC and no pathogenic mutations, 100% of the panelists would avoid the unrestricted indication of bilateral mastectomy. When asked about the possible indications for a bilateral approach, there was also a double consensus for “*under no circumstances*”. Nevertheless, around 70% of the affiliates take family history into consideration when discussing the possibility of a bilateral mastectomy.

In cases of high penetrance germline mutations, almost 100% of the affiliates would consider bilateral mastectomy. For 48.1% of the panelists, bilateral mastectomy should be considered in women ≤60 years of age. Indeed, not only age, but also the affected gene, family history, staging of the disease, safety profile and possibilities of breast reconstruction should be taken into consideration [30, 31]. If indicated, 100.0% of the panelists would use NSM, while < 40% of the respondents would consider BCS.

In cases of patients with TNBC undergoing upfront BCS, 96.8% of the panelists and 83.2% of the affiliates considered “*no ink on tumor*” as being indicative of adequate surgical margins, as previously recommended [32]. The same definition of adequate margins was maintained for BCS following NAT (double consensus).

### Radiotherapy

Affiliates would avoid radiotherapy following mastectomy in patients with T1N0 and T2N0 staging. Among the panelists, however, consensus was only reached following brainstorming (T2N0 from 66.7 to 77.8%). However, 100% of the panelists would indicate adjuvant radiotherapy for T1-2 N1-3 staging, even following NAT and pCR, and for T3N0 patients undergoing mastectomy (double consensus).

According to 92.6% of the panelists, evaluation of flap thickness using imaging methods should be avoided in women with T1-2 N0 tumors submitted to mastectomy. Although some studies have shown a greater amount of terminal duct lobular units and residual disease in flaps > 5.0 mm in thickness [33], no impact was detected on recurrence rates or overall survival rates [34]. Therefore, the consensus was that MRI evaluation of the mastectomy flap should be restricted to situations in which there is some doubt regarding the surgical technique performed.

### Systemic therapy

Most of the affiliates recommended the addition of platinum agents to NAT regimens, irrespective of the presence of germline *BRCA* mutations. Consensus was only achieved among the panelists following brainstorming. The BrighTNess study detected an increase in the rates of pCR and EFS with the addition of carboplatin to the standard regimen of anthracycline and taxane [35], with the gain in pCR occurring irrespective of *BRCA* status [36].

Adding immune checkpoint inhibitors to NAT was recommended by 51.9% of the panelists and by only 30.4% of the affiliates. Nevertheless, consensus was reached among the panelists (81.5%) following discussion on the KEYNOTE-522 study (four cycles of anthracycline-based chemotherapy in a conventional 21-day regimen) [6, 7]. Nevertheless, around 75% of the affiliates recommended that anthracyclines be used in a dose-dense regimen when combining NAC and immunotherapy (double consensus). Following brainstorming, agreement was reversed towards the safety protocol [6, 7].

The presence of germline *BRCA* mutations did not affect the recommendation of NAT with immunotherapy (double consensus). On the other hand, 70.6% of the affiliates considered PD-L1 status to be an indication for neoadjuvant immunotherapy. Among the panelists, this line of thought decreased from 48.1 to 18.1% following discussion, since an increase in the pCR rate was found with the addition of pembrolizumab to NAC, irrespective of PD-L1 expression [6].

Continuing immunotherapy during adjuvant treatment remains controversial [11]. It was recommended that immunotherapy initiated at NAT be continued when there is residual disease (double consensus). However, with pCR, only 45.8% of the affiliates recommended maintaining immunotherapy. The percentage of panelists who agreed with this recommendation increased from 59.3 to 88.9% following brainstorming [6, 7].

For patients with wild-type *BRCA* who had received NAT with immunotherapy and failed to achieve pCR, most affiliates suggested maintaining immunotherapy plus capecitabine. Among the panelists, there was no consensus (51.9% of agreement). This combination of adjuvant therapy has yet to be evaluated in phase III prospective studies, although its safety profile is acceptable in cases of metastatic disease [37]. Conversely, most affiliates disagreed with the adjuvant combination of olaparib and immunotherapy for women with the *BRCA* mutation who achieved pCR following NAT. Despite the reported benefit in disease-free survival with the use of adjuvant olaparib in patients with TNBC and the *BRCA* mutation [9], the combination of iPARP and immunotherapy has yet to be tested as adjuvant therapy.

## Conclusion

Consensus was reached among the experts for > 70% of the questions and agreement between the panelists and the affiliates was moderate. Neoadjuvant chemotherapy should be indicated for almost all cases (except cT1a-b N0) and should include platinum agents. When indicated, immunotherapy is part of the standard of care. The panel reaffirmed the concept of “*no ink on tumor*” as being indicative of adequate margins as well as the possibility of SLNB in cN1 patients who progressed to cN0 following NAT. Nevertheless, one in five of the affiliates still recommended ALND in patients eligible for the ACOZOG Z0011 study. Regarding adjuvant therapy, further prospective studies need to be performed to assess the efficacy and safety of the combination of immunotherapy and capecitabine/olaparib in pertinent cases. Educational interventions affected the panelists’ decision-making in 60% of the questions on systemic treatment, highlighting the relevance of continued education.

## Supplementary Information

**Additional file 1: Table S1.** Profile of the panelists (*n* = 27) and of the SBM affiliated breast surgeons (*n* = 214) who participated in the survey.

**Additional file 2: Table S2.** Summary of the consensus among the panelists prior to brainstorming.

**Additional file 3: Table S3.** Summary of the consensus among the panelists following brainstorming.

**Additional file 4: Table S4.** Comparison of the consensus and agreement between the panelists prior to and following brainstorming.

**Additional file 5: Table S5.** Summary of the consensus among the SBM affiliated breast surgeons.

**Additional file 6: **Table **6.1.** Comparison between the panelists’ decision-making regarding the questions related to diagnosis prior to and following brainstorming. **Table S6.2.** Comparison between the panelists’ decision-making regarding the questions related to surgery prior to and following brainstorming.

**Additional file 7: Table S7.1.** Comparison between the panelists and the SBM affiliated breast surgeons regarding the questions related to diagnosis. **Table S7.2.** Comparison between the panelists and the SBM affiliated breast surgeons regarding the questions related to surgery. **Table S7.3.** Comparison between the panelists and the SBM affiliated breast surgeons regarding the questions related to radiotherapy. **Table S7.4.** Comparison between the panelists and the SBM affiliated breast surgeons regarding the questions related to systemic treatment.

## Data Availability

All data generated or analysed during this study are included in this published article and its supplementary information files.

## Abbreviations

ALND: Axillary lymph node dissection
BCS: Breast-conserving surgery
EFS: Event-free survival
ER: Estrogen and progesterone receptors (ER, PR)
HER-2: Human epidermal growth factor receptor 2 (HER-2)
IHC: Immunohistochemistry
iPARP: Poly adenosine diphosphate-ribose polymerase (PARP) inhibitors
MRI: Magnetic resonance imaging
NAC: Neoadjuvant chemotherapy
NAT: Neoadjuvant therapy
pCR: Pathologic complete response
PR: Progesterone receptors
SBM: Brazilian Society of Mastology
SLN: Sentinel lymph node
TNBC: Triple-negative breast cancer
