# Supplementary material for: Management of early-stage triple-negative breast cancer: recommendations of a panel of experts from the Brazilian Society of Mastology
Source: BMC Cancer. 2022 Nov 22;22:1201. doi: 10.1186/s12885-022-10250-x (PMC9682792; doi:10.1186/s12885-022-10250-x)
Supplement: Supplementary file 1 — Additional file 1: Table S1. Profile of the panelists (n = 27) and of the SBM affiliated breast surgeons (n = 214) who participated in the survey. [file 12885_2022_10250_MOESM1_ESM.docx]

**Table S1:** Profile of the panelists (n=27) and of the SBM affiliated breast surgeons (n=214) who participated in the survey.

| **Characteristics** | **Panelists** | **Affiliated breast surgeons** | **p-value** |
| --- | --- | --- | --- |
| *Mean ± standard deviation* |  |  |  |
| **Age (years)** | 51.74 ± 9.71 | 46.10 ± 10.65 | 0.05 |
| *n (%)* |  |  |  |
| **Sex** |  |  |  |
| Female | 5 (18.5) | 109 (50.9) | **0.01** |
| Male | 22 (81.5) | 105 (49.1) |  |
| **Board certified as breast surgeon** |  |  |  |
| No | 1 (3.7) | 28 (13.1) | 0.18 |
| Yes | 26 (96.3) | 186 (86.9) |  |
| **Region of Brazil** |  |  |  |
| Midwest | 4 (14.8) | 34 (15.9) | 0.32 |
| Northeast | 4 (14.8) | 39 (18.2) |  |
| North |  | 8 (3.7) |  |
| Southeast | 12 (44.4) | 107 (50.0) |  |
| South | 7 (25.9) | 26 (12.1) |  |
| **Works in an academic institution** |  |  |  |
| No | 2 (7.4) | 31 (14.5) | 0.31 |
| Yes | 25 (92.6) | 183 (85.5) |  |
| **Lives in a state capital city** |  |  |  |
| No | 1 (3.7) | 63 (29.4) | **0.04** |
| Yes | 26 (96.3) | 151 (70.6) |  |

*n = absolute frequency; % = relative frequency. SBM: Brazilian Society of Mastology.
