# Supplementary material for: Management of early-stage triple-negative breast cancer: recommendations of a panel of experts from the Brazilian Society of Mastology
Source: BMC Cancer. 2022 Nov 22;22:1201. doi: 10.1186/s12885-022-10250-x (PMC9682792; doi:10.1186/s12885-022-10250-x)
Supplement: Supplementary file 2 — Additional file 2: Table S2. Summary of the consensus among the panelists prior to brainstorming. [file 12885_2022_10250_MOESM2_ESM.docx]

**Table S2:** Summary of the consensus among the panelists prior to brainstorming.

| **Questions** | **Disagreement n (%)** | **Agreement n (%)** | **Item in the subjective questions for which consensus was reached** | **Consensus reached** |
| --- | --- | --- | --- | --- |
| 01 | 11 (40.7) | 16 (59.3) | N/A | No |
| 02 | 11 (40.7) | 16 (59.3) | N/A | No |
| 03 | - | - | N/A | No |
| 04 | 21 (77.8) | 6 (22.2) | N/A | Yes |
| 05 | 8 (29.6) | 19 (70.4) | N/A | Yes |
| 06 | - | - | N/A | No |
| 07 | 0 (0.0) | 27 (100.0) | Neoadjuvant chemotherapy | Yes |
| 08 | - | - | N/A | No |
| 09 | 5 (18.5) | 22 (81.5) | Radiotherapy | Yes |
| 10 | 5 (18.5) | 22 (81.5) | Radiotherapy | Yes |
| 11 | 4 (14.8) | 23 (85.2) | Axillary dissection | Yes |
| 12 | 19 (70.4) | 8 (29.6) | N/A | Yes |
| 13 | - | - | N/A | No |
| 14 | 27 (100.0) | 0 (0.0) | N/A | Yes |
| 15 | 7 (25.9) | 20 (74.1) | Under no circumstances | Yes |
| 16 | 7 (25.9) | 20 (74.1) | N/A | Yes |
| 17 | 0 (0.0) | 27 (100.0) | N/A | Yes |
| 18 | - | - | N/A | No |
| 19 | 0 (0.0) | 27 (100.0) | Nipple-sparing mastectomy | Yes |
| 20 | 25 (92.6) | 2 (7.4) | N/A | Yes |
| 21 | 26 (96.3) | 1 (3.7) | N/A | Yes |
| 22 | 18 (66.7) | 9 (33.3) | N/A | No |
| 23 | 22 (81.5) | 5 (18.5) | N/A | Yes |
| 24 | 0 (0.0) | 27 (100.0) | N/A | Yes |
| 25 | 0 (0.0) | 27 (100.0) | N/A | Yes |
| 26 | 0 (0.0) | 27 (100.0) | N/A | Yes |
| 27 | 17 (63.0) | 10 (37.0) | N/A | No |
| 28 | 1 (3.7) | 26 (96.3) | No ink on tumor | Yes |
| 29 | 2 (7.4) | 25 (92.6) | No ink on tumor | Yes |
| 30 | 3 (11.1) | 24 (88.9) | Clipping or radioactive iodine seed on the tumor prior to chemotherapy | Yes |
| 31 | 1 (3.7) | 26 (96.3) | N/A | Yes |
| 32 | - | - | N/A | No |
| 33 | - | - | N/A | No |
| 34 | 7 (25.9) | 20 (74.1) | N/A | Yes |
| 35 | 11 (40.7) | 16 (59.3) | N/A | No |
| 36 | 11 (40.7) | 16 (59.3) | N/A | No |
| 37 | 13 (48.1) | 14 (51.9) | N/A | No |
| 38 | 14 (51.9) | 13 (48.1) | N/A | No |
| 39 | 22 (81.5) | 5 (18.5) | N/A | Yes |
| 40 | 7 (25.9) | 20 (74.1) | N/A | Yes |
| 41 | 11 (40.7) | 16 (59.3) | N/A | No |
| 42 | 7 (25.9) | 20 (74.1) | N/A | Yes |
| 43 | 13 (48.1) | 14 (51.9) | N/A | No |
| 44 | 18 (66.7) | 9 (33.3) | N/A | No |

*n = absolute frequency; % = relative frequency; N/A = not applicable.

The unspecified questions involve non-Likert-type responses.
