# Supplementary material for: Management of early-stage triple-negative breast cancer: recommendations of a panel of experts from the Brazilian Society of Mastology
Source: BMC Cancer. 2022 Nov 22;22:1201. doi: 10.1186/s12885-022-10250-x (PMC9682792; doi:10.1186/s12885-022-10250-x)
Supplement: Supplementary file 4 — Additional file 4: Table S4. Comparison of the consensus and agreement between the panelists prior to and following brainstorming. [file 12885_2022_10250_MOESM4_ESM.docx]

**Table S4:** Comparison of the consensus and agreement between the panelists prior to and following brainstorming.

| **Questions** | **Kappa coefficient** | **p-value †** | **p-value *** |
| --- | --- | --- | --- |
| **01** | 0.404 | 0.034 | 0.581 |
| **02** | 0.565 | 0.002 | 0.282 |
| **03** | 0.465 | 0.005 | 0.061 |
| **04** | 0.357 | 0.063 | 1.000 |
| **05** | 0.503 | 0.008 | 0.591 |
| **06** | 0.110 | 0.540 | 0.382 |
| **07** | 0.123 | 0.184 | 0.315 |
| **08** | 0.206 | 0.289 | 1.000 |
| **09** | 0.161 | 0.332 | 0.071 |
| **10** | 0.509 | 0.008 | 1.000 |
| **11** | 0.110 | 0.540 | 0.381 |
| **12** | 0.617 | 0.001 | 0.531 |
| **13** | 0.555 | 0.004 | 1.000 |
| **14** | 0.500 | 0.003 | 1.000 |
| **15** | 0.724 | 0.001 | 0.762 |
| **16** | 0.372 | 0.013 | 0.071 |
| **17** | 0.705 | 0.002 | 1.000 |
| **18** | 0.5450 | 0.003 | 0.365 |
| **19** | 0.756 | 0.002 | 1.000 |
| **20** | 0.092 | 0.873 | 0.151 |
| **21** | 0.132 | 0.521 | 0.314 |
| **22** | 0.545 | 0.003 | 0.363 |
| **23** | 0.419 | 0.023 | 0.443 |
| **24** | 0.066 | 0.926 | 0.313 |
| **25** | 0.511 | 0.003 | 1.000 |
| **26** | 0.611 | 0.001 | 1.000 |
| **27** | 0.131 | 0.321 | 0.171 |
| **28** | 0.319 | 0.032 | 0.312 |
| **29** | 0.349 | 0.055 | 0.155 |
| **30** | 0.341 | 0.069 | 0.635 |
| **31** | 0.062 | 0.773 | 0.553 |
| **32** | 0.707 | 0.001 | 0.273 |
| **33** | 0.400 | 0.037 | 0.773 |
| **34** | 0.614 | 0.001 | 1.000 |
| **35** | 0.349 | 0.055 | 0.241 |
| **36** | 0.060 | 0.970 | 0.071 |
| **37** | 0.242 | 0.114 | 0.026 |
| **38** | 0.393 | 0.010 | 0.025 |
| **39** | 0.335 | 0.079 | 0.711 |
| **40** | 0.016 | 0.826 | 0.032 |
| **41** | 0.135 | 0.322 | 0.011 |
| **42** | 0.069 | 0.547 | 0.022 |
| **43** | 0.407 | 0.035 | 1.000 |
| **44** | 0.229 | 0.233 | 0.771 |
| **Total** | 0.465 | 0.005 | 0.171 |

*Chi-square test; †Kappa coefficient.
