# Supplementary material for: Management of early-stage triple-negative breast cancer: recommendations of a panel of experts from the Brazilian Society of Mastology
Source: BMC Cancer. 2022 Nov 22;22:1201. doi: 10.1186/s12885-022-10250-x (PMC9682792; doi:10.1186/s12885-022-10250-x)
Supplement: Supplementary file 5 — Additional file 5: Table S5. Summary of the consensus among the SBM affiliated breast surgeons. [file 12885_2022_10250_MOESM5_ESM.docx]

**Table S5**: Summary of the consensus among the SBM affiliated breast surgeons.

| **Questions** | **Disagreement n (%)** | **Agreement n (%)** | **Item in the subjective questions for which consensus was reached** | **Consensus reached** |
| --- | --- | --- | --- | --- |
| **01** | 81 (37.9) | 133 (62.1) | N/A | No |
| **02** | 50 (23.4) | 164 (76.6) | N/A | No |
| **03** | - | - | N/A | No |
| **04** | 134 (62.6) | 80 (37.4) | N/A | Yes |
| **05** | 85 (39.7) | 129 (60.3) | N/A | No |
| **06** | - | - | N/A | No |
| **07** | 7 (3.3) | 207 (96.7) | Neoadjuvant chemotherapy | Yes |
| **08** | - | - | N/A | No |
| **09** | 58 (27.1) | 156 (72.9) | Radiotherapy | Yes |
| **10** | - | - | N/A | No |
| **11** | 35 (16.4) | 179 (83.6) | Axillary dissection | Yes |
| **12** | 113 (52.8) | 101 (47.2) | N/A | No |
| **13** | - | - | N/A | No |
| **14** | 207 (96.7) | 7 (3.3) | N/A | Yes |
| **15** | 42 (19.6) | 172 (80.4) | Under no circumstances | Yes |
| **16** | 73 (34.1) | 141 (65.9) | N/A | No |
| **17** | 4 (1.9) | 210 (98.1) | N/A | Yes |
| **18** | - | - | N/A | No |
| **19** | 5 (2.3) | 209 (97.7) | Nipple-sparing mastectomy | Yes |
| **20** | 146 (68.2) | 68 (31.8) | N/A | No |
| **21** | 201 (93.9) | 13 (6.1) | N/A | Yes |
| **22** | 156 (72.9) | 58 (27.1) | N/A | Yes |
| **23** | 161 (75.2) | 53 (24.8) | N/A | Yes |
| **24** | 74 (34.6) | 140 (65.4) | N/A | No |
| **25** | 25 (11.7) | 189 (88.3) | N/A | Yes |
| **26** | 21 (9.8) | 193 (90.2) | N/A | Yes |
| **27** | 129 (60.3) | 85 (39.7) | N/A | No |
| **28** | 36 (16.8) | 178 (83.2) | No ink on margin | Yes |
| **29** | 41 (19.2) | 173 (80.8) | No ink on margin | Yes |
| **30** | 54 (25.2) | 160 (74.8) | Clipping or radioactive iodine seed on the tumor prior to chemotherapy | Yes |
| **31** | 6 (2.8) | 208 (97.2) | N/A | Yes |
| **32** | - | - | N/A | No |
| **33** | - | - | N/A | No |
| **34** | 91 (42.5) | 123 (57.5) | N/A | Yes |
| **35** | 105 (49.1) | 109 (50.9) | N/A | Yes |
| **36** | 39 (18.2) | 175 (81.8) | N/A | Yes |
| **37** | 149 (69.6) | 65 (30.4) | N/A | No |
| **38** | 63 (29.4) | 151 (70.6) | N/A | Yes |
| **39** | 120 (56.1) | 94 (43.9) | N/A | No |
| **40** | 53 (24.8) | 161 (75.2) | N/A | Yes |
| **41** | 116 (54.2) | 98 (45.8) | N/A | No |
| **42** | 62 (29.0) | 152 (71.0) | N/A | Yes |
| **43** | 54 (25.2) | 160 (74.8) | N/A | Yes |
| **44** | 104 (48.6) | 110 (51.4) | N/A | No |

SBM: Brazilian Society of Mastology. *n = absolute frequency; % = relative frequency; N/A = not applicable. The unspecified questions involve non-Likert-type responses.
