# Supplementary material for: Management of early-stage triple-negative breast cancer: recommendations of a panel of experts from the Brazilian Society of Mastology
Source: BMC Cancer. 2022 Nov 22;22:1201. doi: 10.1186/s12885-022-10250-x (PMC9682792; doi:10.1186/s12885-022-10250-x)
Supplement: Supplementary file 6 — Additional file 6: Table 6.1. Comparison between the panelists’ decision-making regarding the questions related to diagnosis prior to and following brainstorming. Table S6.2. Comparison between the panelists’ decision-making regarding the questions related to surgery prior to and following brainstorming. [file 12885_2022_10250_MOESM6_ESM.docx]

**Table 6.1:** Comparison between the panelists’ decision-making regarding the questions related to diagnosis prior to and following brainstorming.

| **Question number** | **Panelists** | | **p-value***** |
| --- | --- | --- | --- |
|  | **Before brainstorming**  **n (%)** | **After brainstorming**  **n (%)** |  |
| **Q01** |  |  |  |
| I agree | 16 (59.3) | 14 (51.9) | 0.58 |
| I disagree | 11 (40.7) | 13 (48.1) |  |
| **Q02** |  |  |  |
| I agree | 16 (59.3) | 12 (44.4) | 0.27 |
| I disagree | 11 (40.7) | 15 (55.6) |  |
| **Q03** |  |  |  |
| Only in cases in which neoadjuvant chemotherapy is predicted | 8 (29.6) | 5 (18.5) | **0.04** |
| No | 10 (37.0) | 4 (14.8) |  |
| Yes, in all cases | 9 (33.3) | 18 (66.7) **^‡^** |  |
| **Q04** |  |  |  |
| I agree | 6 (22.2) | 6 (22.2) | 1.00 |
| I disagree | 21 (77.8) | 21 (77.8) |  |
| **Q05** |  |  |  |
| I agree | 19 (70.4) | 17 (63.0) | 0.56 |
| I disagree | 8 (29.6) | 10 (37.0) |  |
| **Q06** |  |  |  |
| No | 4 (14.8) | 2 (7.4) | **0.01** |
| Yes, only from anatomic stage II onwards | 13 (48.1) | 23 (85.2) **^‡^** |  |
| Yes, in all cases | 10 (37.0) **^‡^** | 2 (7.4) |  |
| **Q20** |  |  |  |
| I agree | 2 (7.4) | 0 (0.0) | 0.15 |
| I disagree | 25 (92.6) | 27 (100.0) |  |
| **Q28** |  |  |  |
| No ink on tumor | 26 (96.3) | 27 (100.0) | 0.31 |
| > 2 mm | 1 (3.7) | 0 (0.0) |  |
| **Q29** |  |  |  |
| > 2 mm | 2 (7.4) | 0 (0.0) | 0.15 |
| No ink on tumor | 25 (92.6) | 27 (100.0) |  |
| **Q30** |  |  |  |
| Clipping or radioactive iodine seed on the tumor prior to chemotherapy | 24 (88.9) | 25 (92.6) | 0.60 |
| Performs pigmentation of the skin over the tumor before chemotherapy | 2 (7.4) | 2 (7.4) |  |
| Does not use any kind of marker | 1 (3.7) | 0 (0.0) |  |
| **Q31** |  |  |  |
| I agree | 26 (96.3) | 25 (92.6) | 0.55 |
| I disagree | 1 (3.7) | 2 (7.4) |  |
| **Q32** |  |  |  |
| Mammography | 1 (3.7) | 1 (3.7) | 0.72 |
| Magnetic resonance imaging | 12 (44.4) | 8 (29.6) |  |
| All of the above | 13 (48.1) | 17 (63.0) |  |
| Ultrasonography | 1 (3.7) | 1 (3.7) |  |
| **Q33** |  |  |  |
| Only in cases of partial response or progression | 18 (66.7) | 19 (70.4) | 0.95 |
| No | 8 (29.6) | 7 (25.9) |  |
| Yes | 1 (3.7) | 1 (3.7) |  |
| **Q34** |  |  |  |
| I agree | 20 (74.1) | 20 (74.1) | 1.00 |
| I disagree | 7 (25.9) | 7 (25.9) |  |

*Chi-square test; ^‡^Post hoc; n = absolute frequency; % = relative frequency.

**Table S6.2.** Comparison between the panelists’ decision-making regarding the questions related to surgery prior to and following brainstorming.

| **Question number** | **Panelists** | | **p-value***** |
| --- | --- | --- | --- |
|  | **Before brainstorming**  **n (%)** | **After brainstorming**  **n (%)** |  |
| **Q07** |  |  |  |
| Upfront surgery | 0 (0.0) | 1 (3.7) | 0.31 |
| Neoadjuvant chemotherapy | 27 (100.0) | 26 (96.3) |  |
| **Q08** |  |  |  |
| If T >1 cm | 17 (63.0) | 17 (63.0) | 0.78 |
| If T >2 cm | 7 (25.9) | 8 (29.6) |  |
| If T > 0.5 cm | 2 (7.4) | 2 (7.4) |  |
| In all cases | 1 (3.7) | 0 (0.0) |  |
| **Q09** |  |  |  |
| Axillary dissection | 1 (3.7) | 2 (7.4) | 0.20 |
| Watchful waiting | 4 (14.8) | 9 (33.3) |  |
| Radiotherapy | 22 (81.5) | 16 (59.3) |  |
| **Q10** |  |  |  |
| Axillary dissection | 4 (14.8) | 3 (11.1) | 0.78 |
| Watchful waiting | 1 (3.7) | 2 (7.4) |  |
| Radiotherapy | 22 (81.5) | 22 (81.5) |  |
| **Q11** |  |  |  |
| Axillary dissection | 23 (85.2) | 25 (92.6) | 0.38 |
| Radiotherapy | 4 (14.8) | 2 (7.4) |  |
| **Q12** |  |  |  |
| I agree | 8 (29.6) | 6 (22.2) | 0.53 |
| I disagree | 19 (70.4) | 21 (77.8) |  |
| **Q13** |  |  |  |
| Under no circumstances | 14 (51.9) | 14 (51.9) | 0.77 |
| If < 3 negative lymph nodes are identified | 8 (29.6) | 8 (29.6) |  |
| If only one negative lymph node is identified | 4 (14.8) | 5 (18.5) |  |
| Under all circumstances | 1 (3.7) | 0 (0.0) |  |
| **Q14** |  |  |  |
| I agree | 0 (0.0) | 0 (0.0) | 1.00 |
| I disagree | 27 (100.0) | 27 (100.0) |  |
| **Q15** |  |  |  |
| Under no circumstances | 20 (74.1) | 19 (70.4) | 0.69 |
| Patients < 30 years of age | 2 (7.4) | 1 (3.7) |  |
| Patients < 40 years of age | 5 (18.5) | 6 (22.2) |  |
| Patients < 60 years of age | 0 (0.0) | 1 (3.7) |  |
| **Q16** |  |  |  |
| I agree | 20 (74.1) | 25 (92.6) | 0.07 |
| I disagree | 7 (25.9) | 2 (7.4) |  |
| **Q17** |  |  |  |
| I agree | 27 (100.0) | 27 (100.0) | 1.00 |
| I disagree | 0 (0.0) | 0 (0.0) |  |
| **Q18** |  |  |  |
| In patients < 40 years of age | 0 (0.0) | 1 (3.7) | 0.62 |
| In patients < 50 years of age | 5 (18.5) | 5 (18.5) |  |
| In patients < 60 years of age | 13 (48.1) | 15 (55.6) |  |
| In all cases | 9 (33.3) | 6 (22.2) |  |
| **Q19** |  |  |  |
| Nipple-sparing mastectomy | 27 (100.0) | 27 (100.0) | 1.00 |
| **Q27** |  |  |  |
| I agree | 10 (37.0) | 15 (55.6) | 0.17 |
| I disagree | 17 (63.0) | 12 (44.4) |  |

*Chi-square test; n = absolute frequency; % = relative frequency.

**Table S6.3:** Comparison between the panelists’ decision-making regarding the questions related to radiotherapy prior to and following brainstorming.

| **Question number** | **Panelists** | | **p-value***** |
| --- | --- | --- | --- |
|  | **Before brainstorming**  **n (%)** | **After brainstorming**  **n (%)** |  |
| **Q21** |  |  |  |
| I agree | 1 (3.7) | 0 (0.0) | 0.31 |
| I disagree | 26 (96.3) | 27 (100.0) |  |
| **Q22** |  |  |  |
| I agree | 9 (33.3) | 6 (22.2) | 0.36 |
| I disagree | 18 (66.7) | 21 (77.8) |  |
| **Q23** |  |  |  |
| I agree | 5 (18.5) | 3 (11.1) | 0.44 |
| I disagree | 22 (81.5) | 24 (88.9) |  |
| **Q24** |  |  |  |
| I agree | 27 (100.0) | 26 (96.3) | 0.31 |
| I disagree | 0 (0.0) | 1 (3.7) |  |
| **Q25** |  |  |  |
| I agree | 27 (100.0) | 27 (100.0) | 1.00 |
| I disagree | 0 (0.0) | 0 (0.0) |  |
| **Q26** |  |  |  |
| I agree | 27 (100.0) | 27 (100.0) | 1.00 |
| I disagree | 0 (0.0) | 0 (0.0) |  |

*Chi-square test; n = absolute frequency; % = relative frequency.

**Table S6.4:** Comparison between the panelists’ decision-making regarding the questions related to systemic treatment prior to and following brainstorming.

| **Question number** | **Panelists** | | **p-value***** |
| --- | --- | --- | --- |
|  | **Before brainstorming**  **n (%)** | **After brainstorming**  **n (%)** |  |
| **Q35** |  |  |  |
| I agree | 16 (59.3) | 20 (74.1) | 0.24 |
| I disagree | 11 (40.7) | 7 (25.9) |  |
| **Q36** |  |  |  |
| I agree | 16 (59.3) | 22 (81.5) | 0.07 |
| I disagree | 11 (40.7) | 5 (18.5) |  |
| **Q37** |  |  |  |
| I agree | 14 (51.9) | 22 (81.5) | **0.02** |
| I disagree | 13 (48.1) | 5 (18.5) |  |
| **Q38** |  |  |  |
| I agree | 13 (48.1) | 5 (18.5) | **0.02** |
| I disagree | 14 (51.9) | 22 (81.5) |  |
| **Q39** |  |  |  |
| I agree | 5 (18.5) | 4 (14.8) | 0.71 |
| I disagree | 22 (81.5) | 23 (85.2) |  |
| **Q40** |  |  |  |
| I agree | 20 (74.1) | 12 (44.4) | **0.03** |
| I disagree | 7 (25.9) | 15 (55.6) |  |
| **Q41** |  |  |  |
| I agree | 16 (59.3) | 24 (88.9) | **0.01** |
| I disagree | 11 (40.7) | 3 (11.1) |  |
| **Q42** |  |  |  |
| I agree | 20 (74.1) | 26 (96.3) | **0.02** |
| I disagree | 7 (25.9) | 1 (3.7) |  |
| **Q43** |  |  |  |
| I agree | 14 (51.9) | 14 (51.9) | 1.00 |
| I disagree | 13 (48.1) | 13 (48.1) |  |
| **Q44** |  |  |  |
| I agree | 9 (33.3) | 8 (29.6) | 0.77 |
| I disagree | 18 (66.7) | 19 (70.4) |  |

*Chi-square test; n = absolute frequency; % = relative frequency
