# Supplementary material for: Management of early-stage triple-negative breast cancer: recommendations of a panel of experts from the Brazilian Society of Mastology
Source: BMC Cancer. 2022 Nov 22;22:1201. doi: 10.1186/s12885-022-10250-x (PMC9682792; doi:10.1186/s12885-022-10250-x)
Supplement: Supplementary file 7 — Additional file 7: Table S7.1. Comparison between the panelists and the SBM affiliated breast surgeons regarding the questions related to diagnosis. Table S7.2. Comparison between the panelists and the SBM affiliated breast surgeons regarding the questions related to surgery. Table S7.3. Comparison between the panelists and the SBM affiliated breast surgeons regarding the questions related to radiotherapy. Table S7.4. Comparison between the panelists and the SBM affiliated breast surgeons regarding the questions related to systemic treatment. [file 12885_2022_10250_MOESM7_ESM.docx]

**Table S7.1:** Comparison between the panelists and the SBM affiliated breast surgeons regarding the questions related to diagnosis.

| **Questions** | **Groups** | | **p-value** |
| --- | --- | --- | --- |
|  | **Panelists before brainstorming**  **n (%)** | **Affiliated breast surgeons n (%)** |  |
| **Q01** |  |  |  |
| I agree | 16 (59.3) | 133 (62.1) | 0.77 |
| I disagree | 11 (40.7) | 81 (37.9) |  |
| **Q02** |  |  |  |
| I agree | 16 (59.3) | 164 (76.6) | 0.05 |
| I disagree | 11 (40.7) | 50 (23.4) |  |
| **Q03** |  |  |  |
| Only in cases in which neoadjuvant chemotherapy is predicted | 8 (29.6) | 39 (18.2) | 0.25 |
| No | 10 (37.0) | 73 (34.1) |  |
| Yes, in all cases | 9 (33.3) | 102 (47.7) |  |
| **Q04** |  |  |  |
| I agree | 6 (22.2) | 80 (37.4) | 0.12 |
| I disagree | 21 (77.8) | 134 (62.6) |  |
| **Q05** |  |  |  |
| I agree | 19 (70.4) | 129 (60.3) | 0.31 |
| I disagree | 8 (29.6) | 85 (39.7) |  |
| **Q06** |  |  |  |
| No | 4 (14.8) | 17 (7.9) | 0.36 |
| Yes, only from anatomic stage II onwards | 13 (48.1) | 94 (43.9) |  |
| Yes, in all cases | 10 (37.0) | 103 (48.1) |  |
| **Q20** |  |  |  |
| I agree | 2 (7.4) | 68 (31.8) | **0.01** |
| I disagree | 25 (92.6) | 146 (68.2) |  |
| **Q28** |  |  |  |
| >10 mm | 0 (0.0) | 14 (6.5) | 0.18 |
| >2 mm | 1 (3.7) | 22 (10.3) |  |
| No ink on tumor | 26 (96.3) | 178 (83.2) |  |
| **Q29** |  |  |  |
| >10 mm | 0 (0.0) | 19 (8.9) | 0.23 |
| >2 mm | 2 (7.4) | 22 (10.3) |  |
| No ink on tumor | 25 (92.6) | 173 (80.8) |  |
| **Q30** |  |  |  |
| Clipping or radioactive iodine seed on the tumor  prior to chemotherapy | 24 (88.9) | 160 (74.8) | 0.23 |
| Performs pigmentation of the skin over the tumor  before chemotherapy | 2 (7.4) | 45 (21.0) |  |
| Does not use any kind of marker | 1 (3.7) | 9 (4.2) |  |
| **Q31** |  |  |  |
| I agree | 26 (96.3) | 208 (97.2) | 0.79 |
| I disagree | 1 (3.7) | 6 (2.8) |  |
| **Q32** |  |  |  |
| Mammography | 1 (3.7) | 17 (7.9) | 0.29 |
| Magnetic resonance imaging | 12 (44.4) | 58 (27.1) |  |
| All of the above | 13 (48.1) | 129 (60.3) |  |
| Ultrasonography | 1 (3.7) | 10 (4.7) |  |
| **Q33** |  |  |  |
| Only in cases of partial response or progression | 18 (66.7) | 125 (58.4) | 0.67 |
| No | 8 (29.6) | 75 (35.0) |  |
| Yes | 1 (3.7) | 14 (6.5) |  |
| **Q34** |  |  |  |
| I agree | 20 (74.1) | 123 (57.5) | 0.09 |
| I disagree | 7 (25.9) | 91 (42.5) |  |

SBM: Brazilian Society of Mastology.

*Chi-square test; n = absolute frequency; % = relative frequency.

**Table S7.2:** Comparison between the panelists and the SBM affiliated breast surgeons regarding the questions related to surgery.

| **Questions** | **Groups** | | **p-value*** |
| --- | --- | --- | --- |
|  | **Panelists before brainstorming**  **n (%)** | **Affiliated breast surgeons**  **n (%)** |  |
| **Q07** |  |  |  |
| Upfront surgery | 0 (0.0) | 7 (3.3) | 0.34 |
| Neoadjuvant chemotherapy | 27 (100.0) | 207 (96.7) |  |
| **Q08** |  |  |  |
| In cases of T >1 cm | 17 (63.0) | 116 (54.2) | 0.74 |
| In cases of T >2 cm | 7 (25.9) | 57 (26.6) |  |
| In cases of T >0.5 cm | 2 (7.4) | 24 (11.2) |  |
| In all cases | 1 (3.7) | 17 (7.9) |  |
| **Q09** |  |  |  |
| Axillary dissection | 1 (3.7) | 47 (22.0) **^‡^** | **0.02** |
| Watchful waiting | 4 (14.8) **^‡^** | 11 (5.1) |  |
| Radiotherapy | 22 (81.5) | 156 (72.9) |  |
| **Q10** |  |  |  |
| Axillary dissection | 4 (14.8) | 86 (40.2) | **0.03** |
| Watchful waiting | 1 (3.7) | 9 (4.2) |  |
| Radiotherapy | 22 (81.5) | 119 (55.6) |  |
| **Q11** |  |  |  |
| Axillary dissection | 23 (85.2) | 179 (83.6) | 0.92 |
| Watchful waiting | 0 (0.0) | 1 (0.5) |  |
| Radiotherapy | 4 (14.8) | 34 (15.9) |  |
| **Q12** |  |  |  |
| I agree | 8 (29.6) | 101 (47.2) | 0.08 |
| I disagree | 19 (70.4) | 113 (52.8) |  |
| **Q13** |  |  |  |
| Under no circumstances | 14 (51.9) | 94 (43.9) | 0.70 |
| In all cases | 1 (3.7) | 18 (8.4) |  |
| If <3 negative lymph nodes are identified | 8 (29.6) | 76 (35.5) |  |
| If only 1 negative lymph node is identified | 4 (14.8) | 26 (12.1) |  |
| **Q14** |  |  |  |
| I agree | 0 (0.0) | 7 (3.3) | 0.34 |
| I disagree | 27 (100.0) | 207 (96.7) |  |
| **Q15** |  |  |  |
| Under no circumstances | 20 (74.1) | 172 (80.4) | 0.42 |
| Patients <30 years of age | 2 (7.4) | 16 (7.5) |  |
| Patients <40 years of age | 5 (18.5) | 20 (9.3) |  |
| Patients <60 years of age | 0 (0.0) | 6 (2.8) |  |
| **Q16** |  |  |  |
| I agree | 20 (74.1) | 141 (65.9) | 0.39 |
| I disagree | 7 (25.9) | 73 (34.1) |  |
| **Q17** |  |  |  |
| I agree | 27 (100.0) | 210 (98.1) | 0.47 |
| I disagree | 0 (0.0) | 4 (1.9) |  |
| **Q18** |  |  |  |
| Under no circumstances | 0 (0.0) | 5 (2.3) | 0.13 |
| In patients <40 years of age | 0 (0.0) | 22 (10.3) |  |
| In patients <50 years of age | 5 (18.5) | 45 (21.0) |  |
| In patients <60 years of age | 13 (48.1) | 59 (27.6) |  |
| In all cases | 9 (33.3) | 83 (38.8) |  |
| **Q19** |  |  |  |
| I disagree | 0 (0.0) | 1 (0.5) | 0.67 |
| Nipple-sparing mastectomy | 27 (100.0) | 208 (97.2) |  |
| Simple mastectomy sacrificing nipple-areola complex | 0 (0.0) | 5 (2.3) |  |
| **Q27** |  |  |  |
| I agree | 10 (37.0) | 85 (39.7) | 0.78 |
| I disagree | 17 (63.0) | 129 (60.3) |  |

SBM: Brazilian Society of Mastology.

*Chi-square test; ^‡^Post hoc; n = absolute frequency; % = relative frequency.

**Table S7.3.** Comparison between the panelists and the SBM affiliated breast surgeons regarding the questions related to radiotherapy.

| **Question number** | **Groups** | | **p-value*** |
| --- | --- | --- | --- |
|  | **Panelists before brainstorming**  **n (%)** | **Affiliated breast surgeons**  **n (%)** |  |
| **Q21** |  |  |  |
| I agree | 1 (3.7) | 13 (6.1) | 0.62 |
| I disagree | 26 (96.3) | 201 (93.9) |  |
| **Q22** |  |  |  |
| I agree | 9 (33.3) | 58 (27.1) | 0.48 |
| I disagree | 18 (66.7) | 156 (72.9) |  |
| **Q23** |  |  |  |
| I agree | 5 (18.5) | 53 (24.8) | 0.47 |
| I disagree | 22 (81.5) | 161 (75.2) |  |
| **Q24** |  |  |  |
| I agree | 27 (100.0) | 140 (65.4) | **0.01** |
| I disagree | 0 (0.0) | 74 (34.6) |  |
| **Q25** |  |  |  |
| I agree | 27 (100.0) | 189 (88.3) | 0.06 |
| I disagree | 0 (0.0) | 25 (11.7) |  |
| **Q26** |  |  |  |
| I agree | 27 (100.0) | 193 (90.2) | 0.08 |
| I disagree | 0 (0.0) | 21 (9.8) |  |

SBM: Brazilian Society of Mastology.

*Chi-square test; n = absolute frequency; % = relative frequency.

**Table S7.4:** Comparison between the panelists and the SBM affiliated breast surgeons regarding the questions related to systemic treatment.

| **Question number** | **Groups** | | **p-value*** |
| --- | --- | --- | --- |
|  | **Panelists before brainstorming**  **n (%)** | **Affiliated breast surgeons**  **n (%)** |  |
| **Q35** |  |  |  |
| I agree | 16 (59.3) | 109 (50.9) | 0.41 |
| I disagree | 11 (40.7) | 105 (49.1) |  |
| **Q36** |  |  |  |
| I agree | 16 (59.3) | 175 (81.8) | **0.01** |
| I disagree | 11 (40.7) | 39 (18.2) |  |
| **Q37** |  |  |  |
| I agree | 14 (51.9) | 65 (30.4) | **0.02** |
| I disagree | 13 (48.1) | 149 (69.6) |  |
| **Q38** |  |  |  |
| I agree | 13 (48.1) | 151 (70.6) | **0.02** |
| I disagree | 14 (51.9) | 63 (29.4) |  |
| **Q39** |  |  |  |
| I agree | 5 (18.5) | 94 (43.9) | **0.01** |
| I disagree | 22 (81.5) | 120 (56.1) |  |
| **Q40** |  |  |  |
| I agree | 20 (74.1) | 161 (75.2) | 0.89 |
| I disagree | 7 (25.9) | 53 (24.8) |  |
| **Q41** |  |  |  |
| I agree | 16 (59.3) | 98 (45.8) | 0.18 |
| I disagree | 11 (40.7) | 116 (54.2) |  |
| **Q42** |  |  |  |
| I agree | 20 (74.1) | 152 (71.0) | 0.75 |
| I disagree | 7 (25.9) | 62 (29.0) |  |
| **Q43** |  |  |  |
| I agree | 14 (51.9) | 160 (74.8) | **0.01** |
| I disagree | 13 (48.1) | 54 (25.2) |  |
| **Q44** |  |  |  |
| I agree | 9 (33.3) | 110 (51.4) | 0.07 |
| I disagree | 18 (66.7) | 104 (48.6) |  |

SBM: Brazilian Society of Mastology.

*Chi-square test; n = absolute frequency; % = relative frequency.
